# Caffeine Consumption and Its Potential Health Effects on Saudi Adolescents in Jazan

**DOI:** 10.7759/cureus.44091

**Published:** 2023-08-25

**Authors:** Ebtihal E Eltyeb, Ali A Al-Makramani, Mai M Mustafa, Sahar M Shubayli, Khalid A Madkhali, Shaden A Zaalah, Ali T Ghalibi, Suhaila A Ali, Angum M Ibrahim, Randa A Basheer

**Affiliations:** 1 Faculty of Medicine, Department of Paediatrics, Jazan University, Jazan, SAU; 2 Faculty of Medicine, Jazan University, Jazan, SAU; 3 Faculty of Medicine, Department of Family and Community Medicine, Jazan University, Jazan, SAU; 4 Faculty of Pharmacy, Department of Pharmaceutical Chemistry, Jazan University, Jazan, SAU; 5 Farasan University College, Department of Nursing, Jazan University, Jazan, SAU

**Keywords:** saudi arabia, health effects, consumption, caffeine, adolescent

## Abstract

Background

Caffeine is the most popular product consumed globally in different types and amounts by various age groups. This study aimed to identify caffeine consumption patterns among adolescents in Jazan and explore related health effects.

Methodology

A cross-sectional study was conducted in Jazan on adolescents between 12 and 18 years of age. The consumption of coffee and caffeinated products and their potential health effects were evaluated using a self-reported online questionnaire. The data were analyzed using software for descriptive and inferential statistics.

Results

A total of 718 participants were enrolled in this study, with the age group of 16-18 years constituting the highest percentage (48.9%). Nearly 94% of the participants consumed coffee or caffeinated products, with 57% consuming the products infrequently. About 6.6% consumed coffee or caffeinated products more than three times daily, and about half of the participants used medium-sized cups. The most consumed type of caffeinated beverage was Arabic coffee, followed by tea, soft drinks, and Nescafe. Education level and age group showed a significant correlation with consumption patterns. The most common health effects of caffeine consumption were headaches, irregular sleep, and nausea, which were statistically related to age group, gender, and comorbid conditions.

Conclusions

Consumption of coffee and caffeinated beverages was relatively high among adolescents in this study. Further research on the habits of Saudi adolescent consumers, particularly across different areas of the country, is required.

## Introduction

Caffeine is chemically known as 1,3,7-trimethyl xanthine and is naturally found in many plants, seeds, fruits, nuts, and leaves. In addition to coffee, it is present in varying concentrations in other drinks, such as tea, soft drinks, energy beverages, and chocolate. All these products contain substances belonging to the xanthine family (caffeine, theophylline, and theobromine) [1]. Currently, caffeinated beverages are consumed daily by almost 80% of people worldwide; however, they are consumed differently depending on the type of drink and among different populations [2]. Adolescents often consume caffeine in the form of sweetened coffee and energy drinks, which typically have caffeine concentrations ranging between 70 and 130 mg per 12-ounce serving. This is comparable to an ordinary coffee cup containing about 80 mg of caffeine. However, some bottles of energy drinks exceed the caffeine concentrations commonly found in standard caffeinated soft drinks and are often high enough to cause caffeine toxicity [3,4]. The amount of caffeine in a product can differ significantly based on several variables, such as brand, preparation method, and type of coffee. In the Arab region, Arabian coffee is a popular hot drink that is boiled and served daily on all local social occasions and gatherings. Usually, sugar is not added, and the coffee is consumed with dates and sweets and served in tiny cups.

Although it is generally believed that caffeine in moderate doses (≤400 mg per day) is safe for healthy adults, it is not entirely harmless, as it can cause significant toxicity and even adverse effects that could be fatal at higher doses [5]. As a known antioxidant, coffee can effectively prevent several chronic illnesses, including cancer, diabetes, and heart disease [6]. Moreover, caffeine is a central nervous system stimulant that can increase alertness, decrease weariness, improve performance, and enhance mental function [7,8]. However, higher doses of caffeine (>500 mg per day) pose harmful effects as the quality and duration of sleep are the most frequently noted problems. Moreover, restlessness, nervousness, and gastric pain have been observed with caffeine intoxication [9,10]. The effects of coffee and caffeinated beverages depend on individual characteristics, such as body weight, gender, physiological condition, and the dose consumed [11]. Nevertheless, no study has demonstrated the beneficial or harmful effects on children.

The Kingdom of Saudi Arabia is considered one of the high-income countries where advertising companies actively advertise caffeine products such as soft and energy drinks, causing a high consumption rate in society, in general, and among adolescents, in particular. Therefore, children and adolescents are at greater risk than adults of experiencing adverse effects from caffeine. Despite the high consumption rate of caffeine products in the Saudi community, the consumption of different types of coffee and caffeinated products among Saudi adolescents has not yet been investigated. Therefore, this study aimed to evaluate the consumption of coffee and caffeinated products among adolescents in Jazan and assess the potential associated health effects.

## Materials and methods

A descriptive, cross-sectional study was conducted in Jazan, the capital city of the Jazan region, one of 13 regions in the Kingdom of Saudi Arabia. It is located along the tropical Red Sea coast in southwestern Saudi Arabia. The region is mainly well known for its distinctive (Khawlani) coffee made from trees aged hundreds of years. The area has approximately 1,500 coffee orchards, with an estimated annual production of 270 tons, accounting for 85% of the coffee produced in Saudi Arabia [12].

After obtaining ethical approval from the Institutional Review Board (IRB) of Jazan University (reference number: REC-44/06/475 dated January 5, 2023), a convenience sample from six selected schools was recruited through a self-administrated online questionnaire. The data collection started in January 2023 and lasted until March 2023. The study targeted all students from intermediate and secondary schools in the Jazan region. The inclusion criteria included all male and female students aged 12 to 18 who consented to participate and resided in the Jazan region. It excluded any adolescents who did not meet the inclusion criteria, refused to participate, or did not complete the questionnaire. The sample size was estimated to be 385 using the Cochran formula, n = (z) 2×p(1-p)/d2, where p = 50% is the anticipated response, and z = 95% is the confidence interval, with an error of not more than 4% and a 25% non-response rate. Due to the nature of online surveys, a large sample size is necessary, as the no-response rate is high.

The researchers prepared the questionnaire following guidelines from past studies in the Arab region involving Arabic questionnaires [13]. Experts from the Faculty of Medicine and Pharmacy at Jazan University assessed the questionnaire and offered feedback and suggestions for improvement. The questionnaire was pretested through a pilot study comprising 20 participants to evaluate the effect of the length of the questionnaire on the response pattern and the time taken to complete the survey, as well as to assess the participants’ understanding of the questions. The pretested participants were not included in the final study.

The questionnaire comprised three sections, with a total of 21 questions. The first section consisted of 12 questions that assessed the sociodemographic information of the study participants. The second section consisted of seven questions related to the caffeine consumption habits of the participants (type, amount, and frequency). However, because assessing adolescents’ accurate daily caffeine intake is challenging, we considered caffeine consumption to be a rough estimate of daily intake according to product type and the amount based on canister use or an estimate of cup size typically used. The third section of the questionnaire consisted of two questions to assess the perceived health effects reported by the participants, based on well-known side effects of coffee and caffeinated drinks, and whether they had tried to cease caffeine consumption. The study team distributed the questionnaire to the study participants via school teachers using an anonymous online survey instrument (Google Forms).

The completed questionnaires were reviewed to avoid mistakes. SPSS software version 23.0 (IBM Corp., Armonk NY, USA) was used to analyze data. Initially, all information gathered via questionnaires was coded into variables. Descriptive and inferential statistics involving Pearson’s chi-square test and Fisher’s exact and binary logistic regression test were used to present the results. A p-value of less than 0.05 was considered statistically significant.

## Results

The distributed survey targeted approximately 718 students, with 50% of female students responding. About half of the participants were in the age group of 16-18 years, with nearly 70% of respondents living inside the town. Table 1 shows that most of the participants’ mothers (40%) received a university education compared to 50% of their fathers. About 60% of the participants lived with their small-focus families, and only 29% reported the presence of smokers in their families. As shown in Table 1, the most prevalent chronic illness among the participants was bronchial asthma, followed by anemia and diabetes mellitus.

**Table 1:** Sociodemographic characteristics of the participants (n = 718).

Sociodemographic characteristics		Intermediate school (n = 313)		Secondary school (n = 405)		Total (n = 718)	
Age group	12–13 years	73	23.3%	1	0.2%	74	10.3%
	14–16 years	212	67.7%	81	20%	293	40.8%
	More than 16 years	28	8.9%	323	79.8%	351	48.9%
Gender	Male	88	28.1%	265	65.4%	353	49.2%
	Female	225	71.9%	140	34.6%	365	50.8%
Residence	The rural area around Jazan	79	25.2%	133	32.8%	212	29.5%
	Jazan town	234	74.8%	272	67.2%	506	70.5%
Nationality	Saudi	267	85.3%	354	87.4%	621	86.5%
	Non-Saudi	46	14.7%	51	12.6%	97	13.5%
Mother’s education	Non-educated	29	9.3%	29	7.2%	58	8.1%
	Primary	29	9.3%	30	7.4%	59	8.2%
	Intermediate	52	16.6%	49	12.1%	101	14.1%
	Secondary	78	24.9%	135	33.3%	213	29.7%
	University	125	39.9%	162	40%	287	40.0%
Father’s education	Non-educated	20	6.4%	12	3%	32	4.5%
	Primary	17	5.4%	33	8.1%	50	7.0%
	Intermediate	33	10.5%	35	8.6%	68	9.5%
	Secondary	100	31.9%	96	23.7%	196	27.3%
	University	143	45.7%	229	56.5%	372	51.8%
Economic status	Above average	137	43.8%	151	37.3%	288	40.1%
	Average	146	46.6%	208	51.4%	354	49.3%
	Below the average	30	9.6%	46	11.4%	76	10.6%
Mother’s working status	Working	103	32.9%	148	36.5%	251	35.0%
	Not working	210	67.1%	257	63.5%	467	65.0%
Father’s working status	Working	236	75.4%	302	74.6%	538	74.9%
	Not working	77	24.6%	103	25.4%	180	25.1%
Presence of chronic illness	Thyroid disease	1	1.8%	3	3.0%	4	0.56%
	Cardiac disease	3	5.3%	3	3.0%	6	3.8%
	Renal disease	3	5.3%	5	5.1%	8	0.84%
	Epilepsy	1	1.8%	8	8.1%	9	1.25%
	Diabetes mellitus	5	8.8%	35	35.4%	40	5.8%
	Anemia	25	43.9%	21	21.2%	46	6.4%
	Bronchial asthma	23	40.4%	39	39.4%	62	8.6%
Live with a small family	Yes	188	60.1%	255	63%	443	61.7%
	No	125	39.9%	150	37%	275	38.3%
Live with the extended family	Yes	148	47.3%	171	42.2%	319	44.4%
	No	165	52.7%	234	57.8%	399	55.6%
Presence of smokers in family members	Yes	99	31.6%	112	27.7%	211	29.4%
	No	214	68.4%	293	72.3%	507	70.6%

Table 2 shows that 94% of the study participants consumed coffee or caffeinated drinks; however, as noted in Table 1, there was a small percentage of consumers of coffee or caffeinated drinks in the age group of 12-13 years, which could be a potential source of exaggeration of the total percentage, as younger adolescents may consume less coffee than older.

**Table 2:** Consumption of coffee and caffeinated beverages among the participants.

Variable		Intermediate school (n = 313)		Secondary school (n = 405)		Total (n = 718)	
Drink coffee or caffeinated beverages	Yes	288	92%	390	96.3%	678	94.4%
	No	25	8%	15	3.7%	40	5.6%
Drink coffee with family members	Yes	185	59.1%	235	58%	420	58.5%
	No	128	40.9%	170	42%	298	41.5%
Coffee is only made for visitors	Yes	45	14.4%	78	19.3%	123	17.1%
	No	268	85.6%	327	80.7%	595	82.9%
Drink coffee with friends	Yes	132	42.2%	216	53.3%	348	48.5%
	No	181	57.8%	189	46.7%	370	51.5%
Drink coffee more during the examination period	Yes	82	26.2%	126	31.1%	208	29.0%
	No	231	73.8%	279	68.9%	510	71.0%
Drink coffee because it increases concentration	Yes	106	33.9%	152	37.5%	258	35.9%
	No	207	66.1%	253	62.5%	460	64.1%
Drink coffee because it decreases weight	Yes	26	8.3%	62	15.3%	88	12.3%
	No	287	91.7%	343	84.7%	630	87.7%
Drink coffee to try new types	Yes	110	35.1%	156	38.5%	266	37.0%
	No	203	64.9%	249	61.5%	452	63.0%
Frequency of drinking coffee per day	(n)	(267)		(371)		(638)	
	Once	172	64.4%	240	64.7%	412	64.6%
	2–3 times	77	28.8%	107	28.8%	184	28.8%
	More than three times	18	6.7%	24	6.5%	42	6.6%
The cup size used to drink coffee	(n)	(267)		(371)		(638)	
	Small size	102	38.2%	145	39.1%	247	38.7%
	Medium size	151	56.6%	195	52.6%	346	54.2%
	Large size	14	5.2%	31	8.4%	45	7.1%
Soft drinks and energy drinks consumption	(n)	(288)		(390)		(678)	
	Yes	175	60.8%	212	54.4%	387	57.1%
	No	113	39.2%	178	45.6%	291	42.9%
Frequency of drinking soft drinks and energy drinks	(n)	(175)		(212)		(387)	
	One can daily	46	26.3%	65	30.7%	111	28.7%
	2–3 cans per day	31	17.7%	36	17%	67	17.3%
	More than three cans/per day	6	3.4%	16	7.5%	22	5.7%
	Irregularly	92	52.6%	95	44.8%	187	48.3%
Chocolate consumption	(n)	(288)		(390)		(678)	
	Yes	137	47.6%	132	33.8%	269	39.7%
	No	151	52.4%	258	66.2%	409	60.3%
Frequency of eating chocolate	(n)	(137)		(132)		(269)	
	A chocolate bar/day	27	19.7%	15	11.4%	42	15.6%
	More than 1 bar/day	33	24.1%	35	26.5%	68	25.3%
	Irregularly	77	56.2%	82	62.1%	159	59.1%

About 57% of the participants reported irregularly drinking coffee compared to 28% drinking it daily. While 64% drank coffee or caffeinated beverages once daily, 6.6% drank them more than three times daily. About half of the participants used a medium-sized cup, and 39% used a small cup.

**Figure 1:**
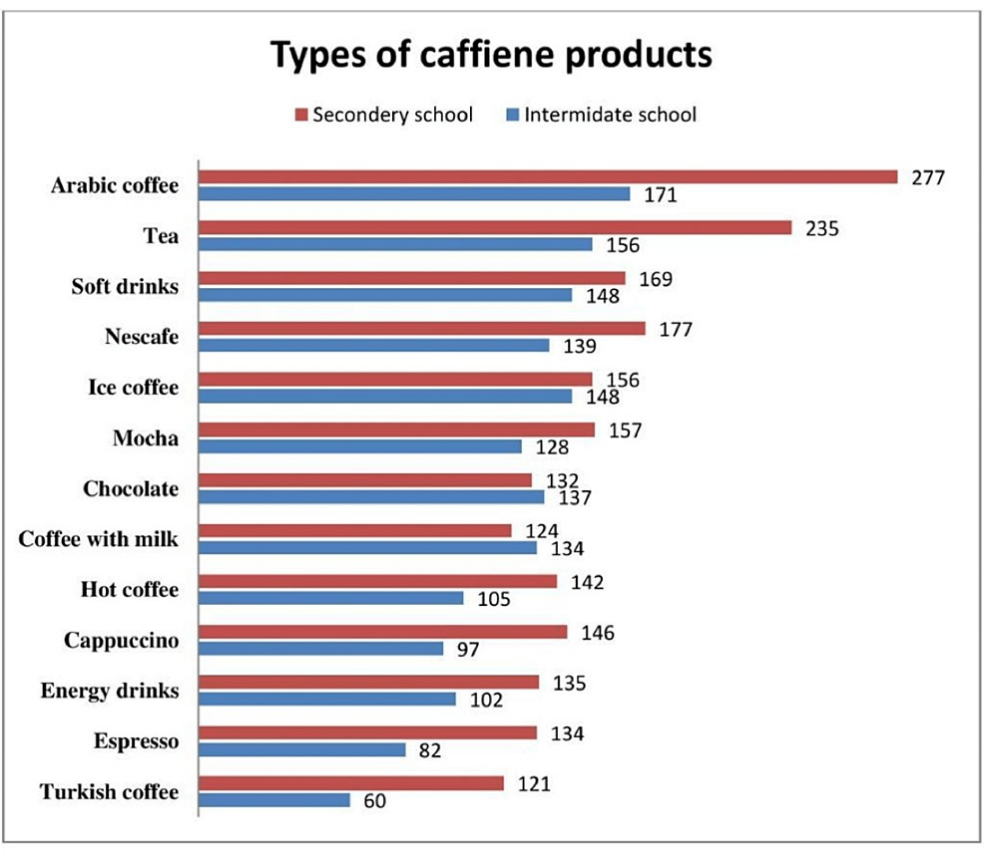
The types of caffeinated beverages consumed by the participants.

Regarding coffee consumption, 58% of the participants drank it with family and 48% with friends. Two-thirds of the participants knew coffee had harmful effects, and 56% thought it had benefits. Figure 1 shows that Arabic coffee was the most frequently consumed caffeinated beverage, followed by tea, soft drinks, and Nescafe. Table 3 shows a significant correlation between the consumption of coffee and caffeinated beverages and the participants’ educational level and age group. Nevertheless, there was no statistical correlation between gender or parental education and the consumption of caffeinated beverages.

**Table 3:** The correlation between the consumption of coffee and caffeinated beverages and other variables. P-values <0.05 are significant.

Variables		Consume coffee or caffeinated beverages		P-value
		Yes	No	
Educational level	Intermediate school	288	25	0.013**
		92%	8%	
	Secondary school	390	15	
		96..3%	3.7%	
Age group	11–13 years	66	8	0.020**
		89.2%	10.8%	
	14–16 years	273	20	
		93.2%	6.8%	
	16–18 years	339	12	
		96.6%	3.4%	
Gender	Male	334	19	0.828*
		94.6%	5.4%	
	Female	344	21	
		94.2%	5.8%	
Residence	The rural area around Jazan	202	10	0.518*
		95.3%	4.7%	
	Jazan town	476	30	
		94.1%	5.9%	
Nationality	Saudi	587	34	0.777*
		94.5%	5.5%	
	Non-Saudi	91	6	
		93.8%	6.2%	
Mother’s education	Non-educated	55	3	0.252*
		94.8%	5.2%	
	Primary	53	6	
		89.8%	10.2%	
	Intermediate	99	2	
		98%	2%	
	Secondary	202	11	
		94.8%	5.2%	
	University	269	18	
		93.7%	6.3%	
Father’s education	Non-educated	28	4	0.384*
		87.5%	12.5%	
	Primary	47	3	
		94%	6%	
	Intermediate	66	2	
		97.1%	2.9%	
	Secondary	184	12	
		93.9%	6.1%	
	University	353	19	
		94.9%	5.1%	
Economic status	Above average	271	17	0.971*
		94.1%	5.9%	
	Average	335	19	
		94.6%	5.4%	
	Below the average	72	4	
		94.7%	5.3%	
Mother’s working status	Working	239	12	0.499*
		95.2%	4.8%	
	Not working	439	28	
		94.00%	6.00%	
Father’s working status	Worked	505	33	0.256*
		93.9%	6.1%	
	Not working	173	7	
		96.1%	3.9%	
Co-morbid illness	Yes	146	10	0.605*
		93.6%	6.4%	
	No	532	30	
		94.7%	5.3%	

**Figure 2:**
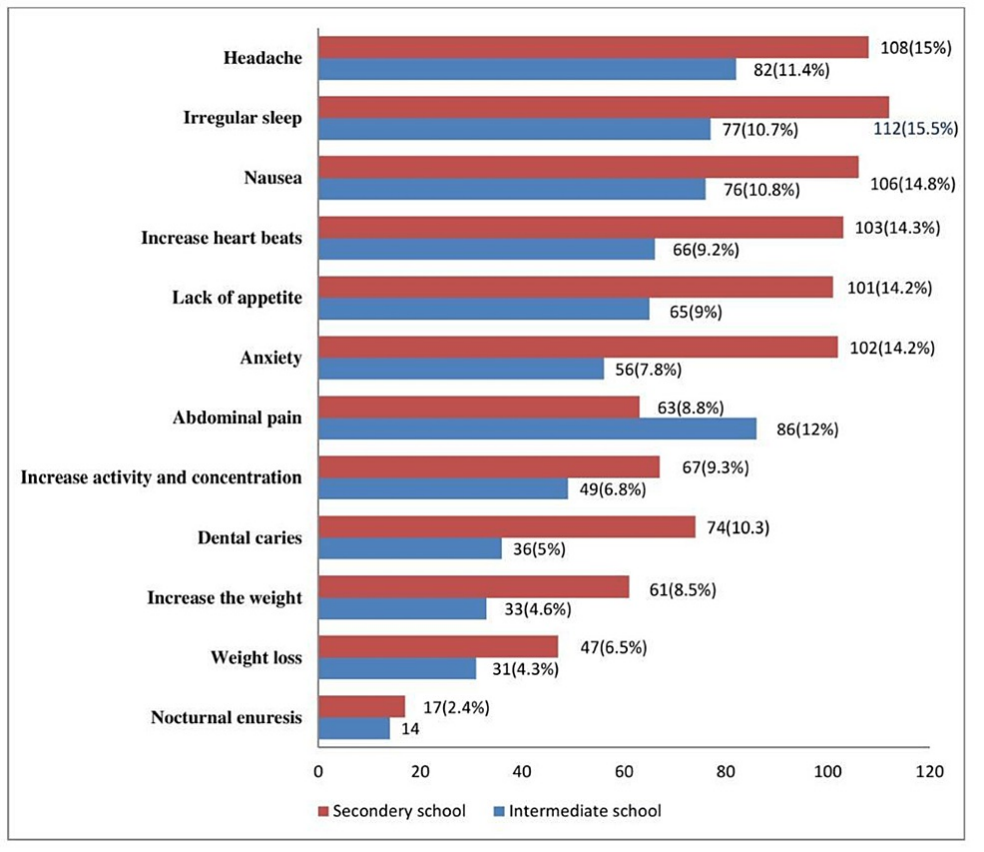
The health effects potentially related to caffeine consumption experienced by the participants.

As reported by the participants, the most frequent health effect related to the consumption of coffee and caffeinated beverages was headache, followed by irregular sleeping and nausea. However, weight loss and nocturnal enuresis were less reported, as shown in Figure 2. The health effects were statistically related to age group, nationality, and chronic illnesses. The binary logistic regression for predicting the health effects of coffee and caffeinated beverages demonstrated the significant relationship between economic status and chronic illnesses and health effects.

## Discussion

In this study, we investigated caffeine intake among adolescents in Jazan City and the possible adverse consequences on their health. To our knowledge, this is the first study of Saudi adolescents using caffeine and a very important study as it is a day-to-day problem that needs attention. The overall prevalence of caffeine consumption in this study was relatively high (94%) when compared to another Saudi study involving female university students (88.2%); however, the prevalence was relatively comparable to a Delhi study (97%) and a Serbian study (99%) that assessed adolescent participants [14-16]. This high prevalence of caffeine consumption can be attributed to two factors. First, the nature of the Arab region as notable consumers compared to other regions of the world, where Arabic coffee and tea are popular hot drinks that are served daily at all social events and local gatherings and reflect the welcoming hospitality. Second, competing caffeine-containing product advertisements routinely shown to adolescents have increased their intake of these drinks and caffeine consumption in recent years [17].

Regarding the type of caffeinated beverage consumed, Arabic coffee was the most frequently consumed type among adolescents (66.1%), followed by tea (57.7%) and soft drinks (46.8%). It is well known that the processing and preparation, including the kind of beverage, temperature, and length of brewing or steeping, influence caffeine concentration. One study compared the caffeine content in Arabic coffee to other types of coffee and reported that the amount of caffeine in one cup of Turkish coffee is equivalent to two Nescafe cups and 20 Arabian cups [18]. Consequently, six cups per day are considered safe for healthy adults; however, no studies have determined the recommended dose for children or adolescents. According to the American Academy of Child and Adolescent Psychiatry, there is no known safe dose of caffeine for children. Pediatricians advise against caffeine in children under 12 years of age and energy drinks for all children and adolescents. For those between the ages of 12 and 18, they advise limiting caffeine intake to no more than 100 mg (or roughly two 12 oz cans of cola) daily [19]. We found that secondary school participants were consuming slightly more than intermediate school participants, indicating that the older the adolescents are, the more caffeine they consume. Accordingly, it is vital to develop awareness, particularly for secondary schools, based on this evidence.

Regarding caffeine consumption habits among adolescents, in this study, about half of the participants consumed caffeinated beverages with friends, and only a third drank them to increase their concentration during the school examination periods. This information reflects peer influences on the consumption habits of adolescents by trying new types of beverages or even increasing the commonness of consumption. Further, data considering the frequency and amount of consumption reported that 28.7% of the participants drank coffee daily, and 54.2% used medium-sized cups. Moreover, adolescents who consumed soft and energy drinks comprised more than half of the participants, with a third consuming them daily. Despite the high prevalence of consumption of caffeinated beverages among the adolescents in this study, it is not easy to calculate the exact daily caffeine intake, frequency of consumption, and contribution to daily fluid intake. The nature of the subjective evaluation method, which relies on participants’ recall, makes accurate estimation difficult. However, in one study, including a group of 12- to 13-year-old Saudi adolescents, it was estimated that 5% (103 mL) of fluid intake was from tea or coffee [20].

One element of our search included investigating the potential adverse consequences of caffeine use on adolescents’ health. In our study, approximately 39.4% of adolescents reported headaches as a possible health effect related to the consumption of coffee and caffeinated beverages. Irregular sleep (39.2%), nausea (37.8%), lack of appetite (34.4%), and anxiety (32.8%) were the most commonly noted other possible health effects by the participants in this study. This result is consistent with the findings reported by a similar study on the health effects of the rate and amount of caffeine consumption, which concluded that children’s consumption should not exceed 2.5 mg of caffeine per kilogram of body weight per day to avoid these related adverse health effects [21]. Consuming too much caffeine can be linked to anxiety, headache, nausea, and restlessness, and caffeine intoxication can result from consuming more than 400 mg, or the amount of caffeine in three to four cups of brewed coffee [22]. The symptoms of caffeine intoxication include disturbed sleep, increased urination, stomach irritability, headache, irregular or rapid heartbeat, and psychomotor agitation [23]. On the other hand, caffeine withdrawal is a well-known trigger of migraine; however, withdrawal symptoms are usually moderate and transient [24-26].

This study is noteworthy because it sheds light on the caffeine consumption of different product types among adolescents. As we observed in school students in the Kingdom of Saudi Arabia, an increased rate contributed to the spread of cafes that sell all cold and hot caffeine products. It was also noted that the prevalence of soft and energy drinks is constantly increasing.

This study provides a preliminary notion of the general situation of caffeine consumption among adolescents. However, several limitations may prevent an accurate assessment of actual consumption due to the methodology, which depends on participants’ remembering and can lead to recall bias. Furthermore, much of the information currently available about the health effects of coffee is derived from personal experiences, regardless of current health status, which reflect subjective rather than objective findings. Therefore, effective evaluation methods are needed to measure the health impact accurately. Consequently, the study of adolescents’ coffee consumption must consider the type and amount of caffeine exposure to rule out potential confounders that could influence the outcome of coffee consumption on their health. Additionally, the impact of co-morbidities or other psychological or physiological factors related to growth and development in this age group may be other confounders that should be considered in the possible health effects reported by adolescents.

## Conclusions

Although several studies have been published on caffeine in various regions of Saudi Arabia, few data are available on Saudi adolescents’ consumption habits. Consumption of coffee and caffeinated beverages was relatively high among adolescents in this study, which may be related to general societal attitudes. Therefore, for an accurate estimation, it is necessary to conduct more studies on caffeine habits, amounts, and health effects on Saudi adolescent consumers in all regions of the country.
